# Zuranolone – synthetic neurosteroid in treatment of mental disorders: narrative review

**DOI:** 10.3389/fpsyt.2023.1298359

**Published:** 2023-12-05

**Authors:** Rafał Marecki, Joanna Kałuska, Agata Kolanek, Dominika Hakało, Napoleon Waszkiewicz

**Affiliations:** Department of Psychiatry, Medical University of Białystok, Białystok, Poland

**Keywords:** zuranolone, SAGE-217, neurosteroid, depression, allopregnanolone, GABA, pregnenolone, brexanolone

## Abstract

With each passing year, the number of people suffering from mental disorders grows at a disturbing speed. Neuroactive steroids are a new promising group of drugs with the potential for use in many diseases like postpartum depression, postnatal psychosis, major depression, insomnia, bipolar disorder, and Parkinson’s tremor, due to their ability to modulate the activity of GABA_A_ receptor. Neurosteroids are progesterone metabolites that are synthesized from cholesterol or steroid hormones in various brain regions. They regulate neuronal development, regeneration, and neurotransmission. They are implicated in mood disorders, anxiety disorders, schizophrenia, PTSD, and impulsive aggression. Neurosteroids have been studied for their potential to prevent or treat neurodegenerative diseases such as Alzheimer’s disease and HIV-associated dementia. They can promote neurogenesis, neuronal survival, myelination, and memory function. They can also affect the growth and sensitivity of hormone-dependent brain tumors such as gliomas. Zuranolone, a newly registered neurosteroid drug has shown huge flexibility in both clinical and ambulatory treatment thanks to its pharmacokinetic traits, especially the possibility for oral administration, unlike its predecessor Brexanolone. Zuranolone is a synthetic positive allosteric modulator of the GABAA receptor that can be taken orally. The review aims to summarize the current knowledge on zuranolone as a novel neurosteroid drug for various mental disorders, especially for postpartum mental disorders for which this drug was meant originally. It covers studies indexed in the PubMed, Scopus, and Web of Science databases published since 2017. Keywords used in the search, as well as inclusion and exclusion criteria, are given in the aims and methodology section. The review explains the evidence for the role of neurosteroids, especially allopregnanolone, in the pathophysiology and treatment of postpartum depression. It discusses the mechanisms of neurosteroid action, the changes in neurosteroid levels during pregnancy and postpartum, and the clinical trials of brexanolone and zuranolone, two synthetic analogs of allopregnanolone, for postpartum depression. It provides an overview of the biosynthesis and metabolism of neurosteroids in the central and peripheral nervous system. Furthermore, it explains the different sources and pathways of neurosteroid production and the factors that influence their synthesis and regulation, such as stress, hormones, drugs, and genetic variations. The review also explores the potential relevance of neurosteroids for other psychiatric disorders, such as major depression, bipolar disorder, post-traumatic stress disorder (PTSD), schizophrenia, and premenstrual dysphoric disorder. Finally, it highlights the associations between neurosteroid levels and symptom severity and the effects of neurosteroid modulation on mood, cognition, and neuroplasticity.

## 1 Introduction

A huge breakthrough in psychiatry was brought about by the development of selective serotonin reuptake inhibitor (SSRI) drugs, but researchers continue their efforts. A new class of therapeutics was developed by the close examination of the neurosteroid system. In 2019, brexanolone was introduced as the first drug containing Allopregnanolone. In 2022 SAGE Therapeutics registered Zuranolone – a novel synthetic neurosteroid. In August 2023 U.S. Food and Drug Administration approved zuranolone (Zurzuvae) in treatment of postpartum psychiatric disorders (PPDs). This drug has been found to have great potential for use in PPDs (1, 2), consequences of which can be tragic without proper treatment. Infanticide and maternal suicide are associated with postpartum depression, and existing treatments often struggle to effectively manage depressive symptoms. Zuranolone was successful in quickly and lastingly alleviating postpartum depression symptoms, and it was well-tolerated by patients (1). It also exhibits very promising effects in the treatment of conditions outside the PPDs group (2–4). In particular, its use has already been reported for treatment of Parkinson’s tremor, insomnia, and bipolar disorder (3, 5–9). Still, its qualities allow one to reason about even wider applications, which could be beneficial in general population. Table 1 briefly summarizes Zuranolone known and possible usage.

**Table 1:** Brief summary of findings gathered in the review.

**Disorders**	**Findings**
**Postpartum depression**	− Zuranolone was found to enhance HAMD-17 scores and alleviate depression symptoms in women with PPD by day 15, compared to a placebo. These effects were noticeable from day 3 and persisted up to 45 days. Additionally, patients reported better anxiety, global, and maternal functioning. In MDD patients, a 14-day zuranolone treatment reduced depressive symptoms as per the HAM-D score (2).
**Major depressive disorder**	− In a double-blind trial with 543 major depressive disorder patients, zuranolone (50 mg daily) showed significant symptom reduction by day 15, with effects noticeable by day 33. The drug was safe, well-tolerated, and presented no new safety concerns (10).
**Insomnia**	− Zuranolone, as a treatment for insomnia, was tested in a study where patients received either a 30 mg or 45 mg dose, or a placebo. Results showed significant improvements in sleep quality and efficiency, with higher doses yielding better outcomes. The treatment increased time spent in N2 and N3 sleep stages, but not in N1 or R stages (5).
**Parkinson’s tremor**	− In an open-label, exploratory study, zuranolone was administered to 14 patients with Parkinson’s disease who were already receiving stable doses of other medications. Zuranolone improved tremor symptoms as early as 12 h following the first dose and was sustained throughout the treatment period. Similar improvements were noted in overall experiences of daily living and medication-related motor complications. Zuranolone was generally well-tolerated, with the most common adverse events being headache and fatigue (9).
**Bipolar disorder**	− Zuranolone has shown promising results in an open-label, phase 2 study of 30 mg daily for 2 weeks in bipolar disorder patients with depressive symptoms. It improved mood, anxiety, and functioning, and was well-tolerated. However, more studies are needed to confirm its efficacy and safety (11).

### 1.1 Background

Pregnancy is a challenging and demanding period for a woman and her family. Emotional stress (thoughts about becoming a mother and worries about a future child’s health), biological factors (hormonal changes), and sleep deprivation can cause perinatal distress. 1-2/1000 women worldwide may develop PPDs in 2-4 weeks following delivery, such as relatively common postpartum depression and anxiety (12). They may also experience rare but more severe postpartum psychosis. It is important not to confuse PPDs with baby blues, which is milder, and does not require medical intervention (12).

#### 1.1.1 Postpartum depression

In diagnosing postpartum depression, the onset of illness is important. The ICD-10 classification states that symptoms must appear within 6 weeks after delivery and codes it as F53.0. On the other hand, DSM-V says that they must develop within 4 weeks after birth but does not differentiate postpartum depression from depression during pregnancy. Both are described as ‘perinatal depression disorders’. It affects a mother’s relationship with an infant and usually her ability to manage day-to-day activities. History of depression, anxiety, sexual abuse, risky pregnancy, lack of social support, and domestic violence are risk factors (13–22).

A rapid change in reproductive hormones like estradiol and progesterone following delivery are potential triggers for susceptible women and can lead to the manifestation of depressive symptoms. Oxytocin and prolactin also play an important role in the pathogenesis. These hormones regulate lactation and the milk let-down reflex. A correlation between lactation failure and the onset of PPD has been found. Low levels of oxytocin are observed in PPD and unwanted early weaning. During the third trimester, lower levels of oxytocin relate to increased depressive symptoms during pregnancy and following delivery (13, 23–26).

The standard treatment for postpartum depression typically includes psychotherapy and antidepressants. However, due to potential risks associated with exposing infants to antidepressants, psychotherapy is often the preferred treatment option for new mothers (27). Interpersonal psychotherapy is particularly relevant for postpartum depression as it focuses on the unique interpersonal challenges faced by women during the postpartum period. Interpersonal psychotherapy operates on the understanding that interpersonal issues arising during pregnancy, coupled with hormonal changes, can lead to depressive symptoms (27). Treatment approaches administered by mental health professionals can positively impact mothers with postpartum depression, leading to improved therapeutic outcomes and psychosocial enhancement. Numerous studies have shown that interpersonal psychotherapy is more effective in treating depression than other psychological treatments (27).

According to statistics up to 4 in 5 new parents (80%) may have baby blues (28). In the days following childbirth (days 1-4), most women experience some transient mental disturbance like mood swings, mild depression, anxiety symptoms, irritability, sleep disturbances, appetite changes, and fatigue that often persist for ≤2 weeks and usually resolve spontaneously with no sequelae (29). The main cause of baby blues is hormonal changes. According to a work by Balaram and Marwaha, estrogen and progesterone levels suddenly decrease after delivery, causing mood swings (29).

#### 1.1.2 Postnatal psychosis

In ICD-10, postnatal psychosis is an affective disorder classified by type (F30-F39) and additionally with code 099.3. DSM-V categorizes it as a ‘*brief psychotic disorder*’ in the section ‘*Disorders in addition to the schizophrenia spectrum and other psychotic disorders.’* Patients may experience hallucinations, insomnia, agitations, and delusions which create a risk of suicide and infanticide. Overall postpartum relapse risk is estimated to be 37% in populations of women with bipolar disorder and 31% with a history of postpartum psychosis (30). The distinction between those two groups is significant for clinical relevance. Patients with bipolar disorder were less likely to experience severe episodes (17%) compared with the second group (29%) (30). Moreover, for women with bipolar disorder, continuation of prophylactic medication during pregnancy appears to be crucial for maintaining mood stability after delivery. In contrast, for women with a history of isolated postpartum psychosis, the initiation of prophylaxis immediately after the delivery was found to be highly effective for relapse prevention and eliminates the risk of *in-utero* medication exposure (30–36).

Pharmacological intervention for postpartum psychosis has not been the focus of many recent studies. This could be due to the already proven effectiveness of lithium, along with supplementary antipsychotics, benzodiazepines, and electroconvulsive therapy in treating postpartum psychosis (34). There is a lack of information on the application of newer antipsychotics (cariprazine, lumateperone, brexpiprazole, pimavanserin) (34). Despite the progress in neurosteroid treatments for postpartum depression with brexanolone, no research has assessed its potential use in postpartum psychosis (34).

#### 1.1.3 Severe depression

Depression is a highly prevalent disorder with a lifetime prevalence of about 12%. It is more common in women than in men and in younger adults compared to older people. It is often comorbid with other psychiatric and medical disorders and increases the risk of suicide (28). To diagnose, a patient needs to present with 5 or more of any symptoms nearly every day for a 2-week period, provided at least one of these symptoms is a fundamental one. Symptoms of MDD can be decreased mood, anhedonia, feelings of worthlessness or guilt, suicidal ideation, planning or attempt, fatigue or loss of energy, insomnia or hypersomnia, loss or increase in appetite, and significantly decreased cognitive functions (29). Major depression is a common illness that severely limits daily functioning and diminishes the quality of life. In 2008, WHO ranked major depression as the third highest burden of disease worldwide and predicts that it will rank first by 2030 (37). Across the lifespan, depression is almost twice as common in women than in men (38).

### 1.2 Neurosteroids

Neurosteroids are a group of cholesterol metabolites, which naturally occur in mammals’ nervous systems. Despite their origin, they do not activate peripheral receptors for steroid hormones. Instead, they regulate the depolarization of neuronal cells by modulating membrane receptors (39).

Proneurosteroids are steroid hormones that are used for the further synthesis of neurosteroids. Examples are dihydrotestosterone and testosterone which are substrates for synthesis of inhibitory positive or negative modulators of GABA_A_ receptors from the androstenes group. Furthermore, there is a group of pregnanes that comes from pregnenolone and progesterone, which act in the same manner (39). Neurosteroids belonging to these two categories can have inhibitory or excitatory effects on CNS. In the case of this study, the most important are allopregnanolone, its isomers, DHEA, and S-DHEA.

Dehydroepiandrosterone (DHEA) and its sulfated variant DHEA-S are the most prevalent steroids in circulation and serve as precursors to active sex hormones like estradiol and testosterone. They have a wide array of effects on various systems including the central nervous system, cardiovascular system, adipose tissue, kidney, liver, and reproductive system (40). The biological effects of DHEA and DHEA-S are initiated through diverse mechanisms. They can directly bind to plasma membrane receptors such as a DHEA-specific G-protein coupled receptor in endothelial cells and various neuroreceptors like GABA(A), NMDA, and sigma-1 receptors (40). They can also bind directly to nuclear androgen and estrogen receptors, albeit with lower binding affinities compared to steroid hormones like testosterone, dihydrotestosterone, and estradiol. The neuroprotective roles of DHEA and DHEA-S are associated with the inhibition of GABA_A_ and the activation of NMDA (40).

Allopregnanolone and its stereoisomers pregnanolone, epipregnanolone, and isopregnanolone are all allosteric modulators of the GABA_A_ receptor. Iso- and epipregnanolone act as negative modulators, whereas allopregnanolone and pregnanolone modulate the receptor positively (39). Clinically, a change in their serum and CSF concentrations proportion is observed in correlation with certain mental disorders like PPDs (41). The available publications indicate a relative decrease in concentration of a positively modulating isomer in comparison to the concentration of a negatively modulating isomer. Physiologically, fluctuations of neurosteroids are related to ovarian cycle in females, and in males, there is a constant decrease in concentration related to aging (42).

#### 1.2.1 Synthesis

The term “*neurosteroids*” was coined to describe steroids produced by the nervous system when studies provided evidence suggesting that DHEA sulfate could be synthesized endogenously in the brain, independent of secretions from the adrenals and testes (42). However, it is crucial to note that more precise measurements, such as those obtained through mass spectrometry techniques, later indicated that pregnenolone or DHEA sulfates in rodents should not be included in the concept of neurosteroids (42). Subsequent studies revealed the expression of the enzyme P450 side chain cleavage in the brain. This enzyme plays a key role in the initial step of steroidogenesis, converting cholesterol into pregnenolone. This conversion was observed in oligodendrocytes, thereby confirming the existence of neurosteroids (42). Steroidogenesis occurs both in the nervous system and peripheral glands, and it is crucial to understand how these two steroid sources interact (42).

A high concentration of neuroactive steroids is found in central nervous system (CNS), much higher than in serum. Research shows that CNS have high expression of enzymes responsible for steroidogenesis. The majority present in gonads and adrenal glands is also present in CNS. It uses both steroid hormones crossing blood–brain barrier (BBB) and those synthesized *de novo* from cholesterol for further transformation into neurosteroids (43, 44).

Neurosteroidogenesis begins in mitochondria when they receive cholesterol. This transfer involves steroidogenic acute regulatory protein (StAR) and translocator protein 18 kDa (TSPO). Next step is a transformation of cholesterol into pregnenolone with the involvement of the mitochondrial side-chain cleavage enzyme (P450scc). Then pregnenolone is directed to the progesterone path or 17OH-pregnenolone path, which is further converted to DHEA. Both paths converge, as progesterone and DHEA are used for the synthesis of androstenedione, and then testosterone. Testosterone is transformed to 17β-estradiol by an enzyme called aromatase. Research shows that regions such as cortex, hippocampus, hypothalamus, and cerebellum express high steroidogenic activity. Also, gender-specific differences are observed (45, 46). Table 2 Indicates the localization of synthesis of certain steroids in the central nervous system.

**Table 2:** Localization of synthesis of certain steroids in central nervous system.

Type of cells	Produced steroids (43)
Neurons	Pregnenolone DHEA Androstenedione Estrogens
Astrocytes	Pregnenolone Progesterone DHEA Androstenedione Testosterone Estradiol Estrone
Oligodendrocytes	Pregnenolone Progesterone Androstenedione
Microglia	5-androstene-3β 17β-diol (ADIOL)

#### 1.2.2 Normal brain economy of neuroactive steroids in mice

In both young adult male and female mice, substantial amounts of progesterone, 5α–dihydroprogesterone, and allopregnanolone were detected in the brain. It was demonstrated in female mice that the brain concentrations of progesterone and 5α–dihydroprogesterone fluctuated significantly with the estrus cycle. As a result, depending on the cycle phase, the brain progesterone levels were either comparable to or significantly higher in females than in males. However, there was no significant difference in the brain levels of allopregnanolone between males and females (47).

Aging is linked to a reduction in the production of steroids. A previous study has examined the pattern of progesterone and its by-products in the brains of young (3-month-old) and old (20-month-old) male and female mice. Its findings indicate a significant reduction in the brain concentrations of progesterone, 5α–dihydroprogesterone, and allopregnanolone in older mice of both genders. Notably, the gender differences seen in young mice were not present in the older mice (47). Table 3 shows concentrations of neuroactive steroids in mice brain hemispheres depending on sex and ovarian cycle phase which comes from research conducted by Gaignard et al. (48).

**Table 3:** Levels of neuroactive hormones in mice in ng/g.

	**Group**	**Pregnenolone**	**Progesterone**	**5α-dihydroprogesterone**	**Allopregenanolone**	**Isoprenennolone**
**ng/g**						
(48)	3-month-old mice	Males: 5.59 ± 0.71 Females (diestrus): 11.56 ± 1.06	Males: 2.46 ± 1.10 Females (diestrus): 35.08 ± 5.79	Males: 19.22 ± 2.13 Females (diestrus): 20.39 ± 2.24	Males: 1.05 ± 0.46 Females (diestrus): 2.39 ± 0.42	Males: 0.21 ± 0.05 Females (diesteus): 0.29 ± 0.04
	3-month-old female mice	Proestrus: 10.88 ± 0.83 Estrus: 13.47 ± 1.89 Diestrus: 12.02 ± 1.23	Proestrus: 9.01 ± 4.63 Estrus: 4.24 ± 2.74 Diestrus: 30.14 ± 3.87	Proestrus: 7.52 ± 2.20 Estrus: 3.63 ± 1.31 Diestrus: 20.90 ± 2.82	Proestrus: 3.29 ± 1.24 Estrus: 2.10 ± 1.02 Diestrus: 2.53 ± 0.50	Proestrus: 0.27 ± 0.12 Estrus: 0.15 ± 0.05 Diestrus: 0.25 ± 0.02
	20-month-old mice	Males: 4.89 ± 0.28 Females: 3.46 ± 0.50	Males: 0.28 ± 0.10 Females: 1.30 ± 0.50	Males: 2.85 ± 0.43 Females: 3.79 ± 0.40	Males: 0.63 ± 0.25 Females: 1.10 ± 0.47	Males: 0.12 ± 0.04 Females: 0.05 ± 0.02

#### 1.2.3 Post-mortem investigation of the human female brain

The levels of progesterone, 5α-dihydroprogesterone, and allopregnanolone after death were examined in 17 different brain regions and in the serum of five women of reproductive age and five postmenopausal women (49). The study found that there were regional differences in the brain concentrations of all three steroids they were investigating. Furthermore, the concentrations of these steroids were significantly higher in fertile women in the luteal phase of their menstrual cycle compared to postmenopausal controls (49).

The levels of progesterone in a woman’s brain appear to be connected to her endocrine status. This is evident from the fact that progesterone concentrations in fertile women are significantly higher than those in postmenopausal women (49). Additionally, the correlation between progesterone levels in the serum and brain tissue suggests that serum levels significantly influence the brain’s absorption of progesterone (49).

The study discovered that the regional distribution of certain steroids differs from that of progesterone. A notable observation was the relationship between progesterone and 5α–dihydroprogesterone, as well as 5α–dihydroprogesterone and allopregnanolone brain concentrations. However, there was no correlation found between progesterone and allopregnanolone concentrations (49). This suggests that the conversion of progesterone to the powerful neurosteroid allopregnanolone occurs in two distinct stages. The first enzyme, 5α-reductase, is located in the neurons, while the second enzyme, 3α-hydroxysteroid dehydrogenase, is found in the astrocytes (49).

#### 1.2.4 Role of neurosteroids in psychopathology

Certain 3-alpha-reduced neuroactive steroids such as allopregnanolone (3α,5α-tetrahydroprogesterone; 3α,5α-THP) and pregnanolone (3α,5β-tetrahydroprogesterone; 3α,5β-THP) are positive allosteric modulators of the GABA_A_ receptor complex (50). Stimulation of these receptors results in decreased anxiety, sedation, and decrease in seizure activity (45, 46, 51–59).

Patients with major depression, anxiety disorders, premenstrual dysphoric disorder (60), negative symptoms of schizophrenia and impulsive aggression are observed to have decreased levels of allopregnanolone both in the serum and CSF (50, 61–64).

Allopregnanolone and pregnanolone decrease, while Isopregnanolone (3β,5α-tetrahydroprogesterone; 3β,5α-THP) levels increase in major depression and different antidepressants can bring this imbalance back to equilibrium (65–67). Research show that 3β,5α-THP acts as an antagonist for those 3α-reduced neurosteroids (68).

A similar imbalance was recognized in a population of premenopausal women with PTSD. They have significantly lower levels of allopregnanolone in CSF. This results in an increase in PTSD episode frequency as well as the frequency of depressive symptoms (69–73).

During development, neurosteroids take part in neuronal modelling. Dehydroepiandrosterone and dehydroepiandrosterone sulphate stimulate embryonic axonal and dendritic growth, respectively (74). Allopregnanolone stimulates neurite regression. Neuroactive steroids, as mentioned, modulate neurotransmitter receptor expression. Researchers suggest these qualities could be used to promote neurogenesis, neuronal survival, myelination, and improve memory (51, 75–83).

Patients with adrenal insufficiency (AI) experience a significant and premature loss of DHEA production, making them a model for isolated DHEA deficiency. Studies have shown that treatment with DHEA can restore DHEA, androstenedione, and testosterone levels to the normal range in AI patients. It also leads to decreased levels of sex hormone-binding globulin (SHBG), total cholesterol, and high-density lipoprotein (HDL) cholesterol. DHEA replacement therapy significantly improves overall well-being, particularly by reducing depression, anxiety, and fatigue. It also enhances certain aspects of sexual function and satisfaction. There is increasing acceptance that DHEA replacement therapy may be beneficial for a significant percentage of patients with adrenal insufficiency (84).

#### 1.2.5 Role of metabolism of neurosteroids in neurodegenerative diseases

The other research have highlighted the potential of neuroactive steroids as therapeutic option for neurodegenerative diseases, such as Alzheimer’s disease (AD), due to translocator protein (TSPO) and 17β-hydroxysteroid dehydrogenase type 10 (17β-HSD10) have been studied in relation to their mitochondrial dysfunction and neurosteroidgenesis (85). 17β-HSD10 is an enzyme found in the mitochondria that is involved in the metabolism of steroid hormones. This enzyme is critical for neurosteroidogenesis and the breakdown of isoleucine. Genetic mutations in 17β-HSD10 have been linked to delayed brain development and dysfunction. While it is a vital enzyme for the survival of neurons in a healthy brain, the research have suggested that 17β-HSD10 could be a potential therapeutic target and biomarker for AD (85).

Studies have shown that reduced levels of allopregnanolone in the prefrontal cortex are inversely correlated with the stage of the neuropathological disease. Decreased plasma levels of allopregnanolone have also been observed in individuals in the early stage of AD. Elevated glial TSPO expression has been detected early in the disease process and corelates with neuropathology, while increased expression of 17β-HSD10, while increased expression of 17β-HSD10 is found in activated astrocytes. These findings suggest potential treatment opportunities for AD patients (86).

#### 1.2.6 Role of DHEA in aging

As previously mentioned, DHEA takes part in neuronal modelling. Its secretion declines with age, and it has sparked interest as potential “anti-aging” hormone. However, it is unclear whether the age-related decline in DHEA secretion represents a harmful deficiency (84).

Due to the age-related decline in circulating DHEA levels, several randomized trials have investigated the effects of oral DHEA supplementation in healthy elderly individuals. Unfortunately, most of these studies have not shown any benefits of DHEA on well-being, mood, or cognition. One possible explanation for this lack of efficacy is selection bias, as most studies included only healthy individuals with excellent baseline performance, limiting the potential for further improvement. There Neurosteroids in dementia and aging is still a need for broader studies to explore the effects of DHEA supplementation in the elderly (84).

Currently, there is no established indication or widely accepted pharmacological preparation of DHEA for treatment. However, the current evidence on the age-related decline in circulating DHEA levels in healthy individuals does not justify routine DHEA supplementation (84).

#### 1.2.7 Role of allopregnanolone in aging

Research from 2020 of the regulation and levels of allopregnanolone, as well as the impact of allopregnanolone supplementation on cognitive function in mice revealed a decrease in the expression of enzymes involved in the allopregnanolone synthesis pathway and an increase in corticosterone synthesis. Allopregnanolone supplementation was found to enhance cognitive function. When young animals were infused with interleukin 6 (IL-6), there was a significant decrease in allopregnanolone production compared to controls (87). Notably, blocking IL-6 with its natural inhibitor, soluble membrane glycoprotein 130, significantly improved spatial memory in older mice. Results suggest that an age-related increase in IL-6 levels leads to a decrease in the availability of the progesterone substrate, resulting in a decline in allopregnanolone levels and an increase in corticosterone (87). Moreover, findings indicate that allopregnanolone serves as a crucial link between inflammatory cytokines and cognitive decline associated with aging.

Neurosteroids, including allopregnanolone, play a role in regulating regeneration and repair systems in the brain. Allopregnanolone has been extensively studied for its potential to promote regeneration in both the central and peripheral nervous systems. Research on preclinical models of aging and Alzheimer’s disease has provided insights into the regenerative effects of allopregnanolone and its ability to reduce Alzheimer’s pathology (88).

In the brain, allopregnanolone has been found to stimulate the generation and survival of new neurons in the hippocampus of aged mice and mice with Alzheimer’s disease. This regeneration is accompanied by the restoration of associative learning and memory function. The therapeutic efficacy of allopregnanolone was observed in both normal aging and Alzheimer’s disease models, with dosage and treatment regimens influencing its effectiveness. Therefore, allopregnanolone serves as an example of therapeutics that target endogenous regeneration and can determine the success of regeneration-based therapies (88).

#### 1.2.8 Neurosteroids in HIV-associated dementia

Human immunodeficiency virus (HIV) accelerates the production and destruction of CD4+ T lymphocytes, and it results in acquired immunodeficiency syndrome (AIDS) (89). Even though antiretroviral therapy has been administrated successfully, neuronal complications occur, causing cognitive, motor and autonomic impairments. This is classified as HIV-associated dementia (HAD). It poses a significant burden on the healthcare system in terms of treatment and patient care (90). Therefore, it is crucial to identify new targets and drugs for the treatment of HAD.

The research showed that cognitive decline observed in HAD is primarily caused by a widespread reduction in synaptic connections rather than neuronal loss. However, the precise cause of neurotoxicity in HAD is the activation of NMDA receptors by HIV proteins. The neuroprotective roles of DHEA and DHEA-S are associated with the activation of the N-methyl-d-aspartate receptors (NMDARs), a large heterotetrameric group of ligand-gated ionotropic glutamate receptors, facilitate excitatory postsynaptic signaling in the central nervous system (40). These receptors bind neurotransmitters and allosteric effectors, controlling the transmembrane Mg^2+^ and Ca^2+^ ion channels that play a crucial role in neuronal physiology, synaptic plasticity, and neuropsychiatric disorders (40). DHEA and DHEA-S function as allosteric effectors that enhance NMDAR signaling (40), which can be used as new targets in the treatment of HAD. Neuroactive steroids, as mentioned, play a vital role in neuronal branching, forming synaptic connections, and myelinizations during organogenesis (91).

Pregnanolone is found to have a potential protective effect over neuronal apoptosis against glutamate in the hippocampal neurons (92). Pregnenolone at a concentration of 500 nM has been found to protect HT-22 cells from the harmful effects of glutamate. When HT-22 cells were exposed to glutamate, there were noticeable alterations in the overall cellular structure of hippocampal neurons, as demonstrated by cell lysis. However, pretreatment with pregnenolone almost entirely prevented these morphological changes, and the cells resembled untreated control cells (92). The immunofluorescence patterns of glucocorticoid receptors (GR) showed that untreated control cells had less nuclear localization of GR, as determined by the intensity of immunofluorescence. HT-22 cells treated with 5 mM glutamate for 20 h showed a significant increase in GR visualization. However, treatment with 500 nM pregnenolone for 24 h, followed by 5 mM glutamate for 20 h, significantly reduced the nuclear localization of GR (92). The relative nuclear to cytoplasmic fluorescence ratio was calculated to be 0.07, 0.08, 1.52, and 0.14 in control, pregnenolone alone treated, glutamate treated, and pregnenolone followed by glutamate-treated cells, respectively (92). This suggests that pregnenolone treatment can modulate the cellular response to glutamate toxicity (92).

Synaptic loss and neuronal cell death in HAD can also be attributed to the loss of microtubule-associated protein 2 (MAP-2, a protein important for neuronal structure) caused by nitric oxide (NO) and the accumulation of cholesterol in mitochondria. Since the inhibition of pregnenolone synthesis inevitably leads to MAP-2 loss, it would be worthwhile to conduct studies exploring the potential of neurosteroids to reverse or reduce synaptic loss. By gathering further evidence, supplementing neurosteroids alongside highly active antiretroviral therapy (HAART) could be considered as a potential treatment strategy for this significant disease (93).

#### 1.2.9 Role of neurosteroids in neoplasm

Neurosteroids play a potential role in the treatment and development of gliomas, particularly astrocytoma, which are hormone-sensitive brain tumors. The incidence of glioblastoma (GBM) in adults is higher in men compared to women. Female patients also tend to have a better prognosis than male patients with GBM (94).

Researchers have focused on unravelling the molecular mechanisms by which estrogens, progestogens, and androgens impact glioma genesis. However, the complexity arises from the discovery of multiple isoforms of membrane and nuclear steroid receptors, including estrogen receptors. Further research will shed light on signaling pathways and identify downstream targets affected by steroids in astrocytoma (43).

The other findings highlight the involvement of neurosteroids in glioblastoma and suggest that the inhibition of neurosteroid synthesis could be a potential therapeutic approach. The study provides insights into the complex pathways of neurosteroid synthesis in GBM cells and the potential use of enzyme inhibitors like finasteride and dutasteride to modulate neurosteroid production (94).

Study revealed the presence of steroidogenic acute regulatory protein (StAR) in oligodendrogliomas (ODs), particularly in low-grade tumors and suggested that the neurosteroidgenesis mechanism mediated by StAR may play a role in the growth of ODs. The authors found that low-grade ODs exhibited gene expression patterns distinct from high-grade ODs and normal brain tissue. Genes involved in cell cycle, DNA replication, migration, protein modification, and signal transduction were upregulated as the tumor grade increased. The high expression of genes related to lipid and steroid metabolism in ODs opens possibilities for developing targeted therapies that exploit these metabolic pathways (95).

Findings suggest that neurosteroids can have both pro-tumorigenic and anti-tumorigenic effects, depending on various factors, including the specific neurosteroid, concentration, duration of exposure, and the cancer type. Further research is needed to fully understand the mechanisms underlying the effects of neurosteroids on cancer and to explore their potential as therapeutic agents in cancer treatment.

## 2 Aims and methodology

Postpartum psychiatric disorders, bipolar disorders, major depression, insomnia and Parkinson’s tremors significantly decrease quality of life. A search for newer methods is vital to provide the best treatment to everyone. This study aims to review zuranolone – a new drug used for the aforementioned disorders.

**Figure 1:**
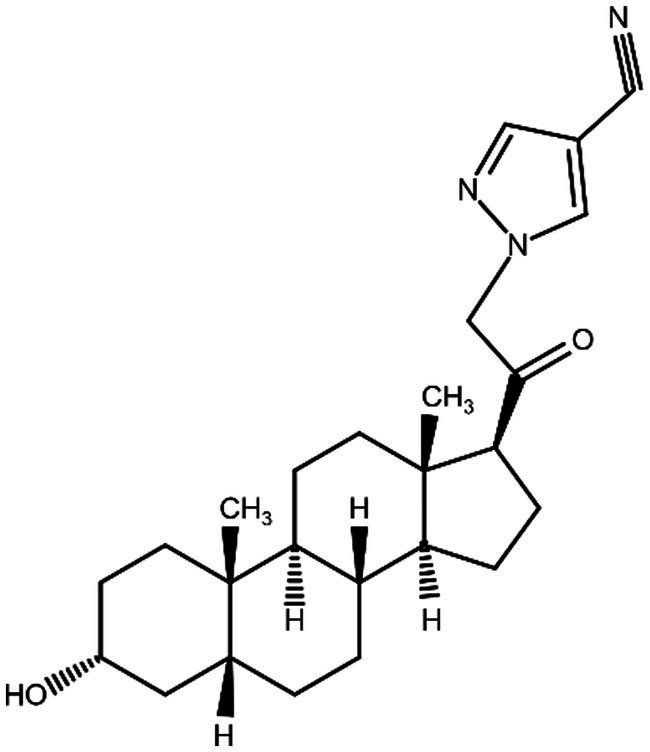
Zuranolone (SAGE-217).

**Figure 2:**
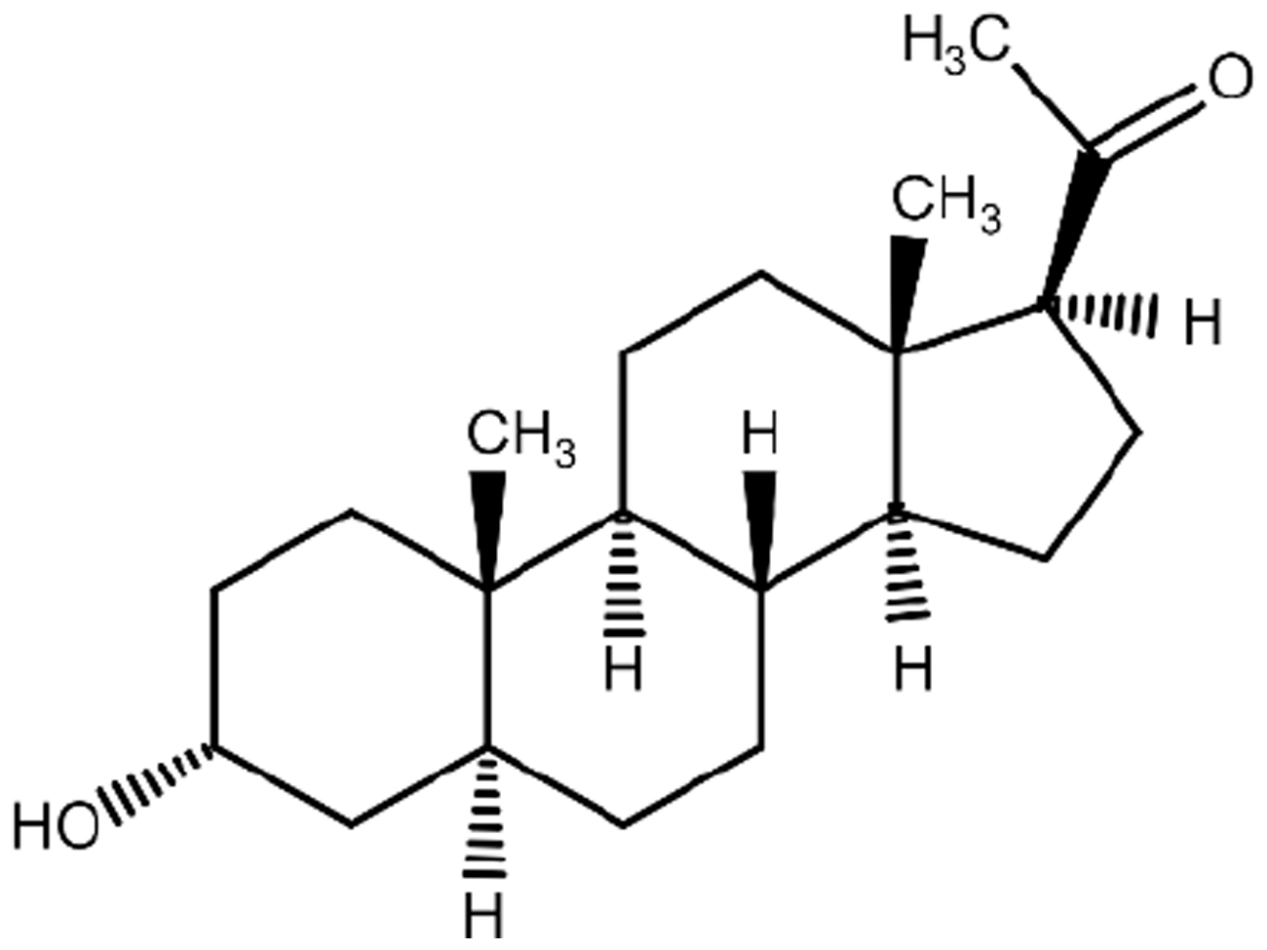
Allopregnanolone (Brexanolone) chemical structure.

**Figure 3:**
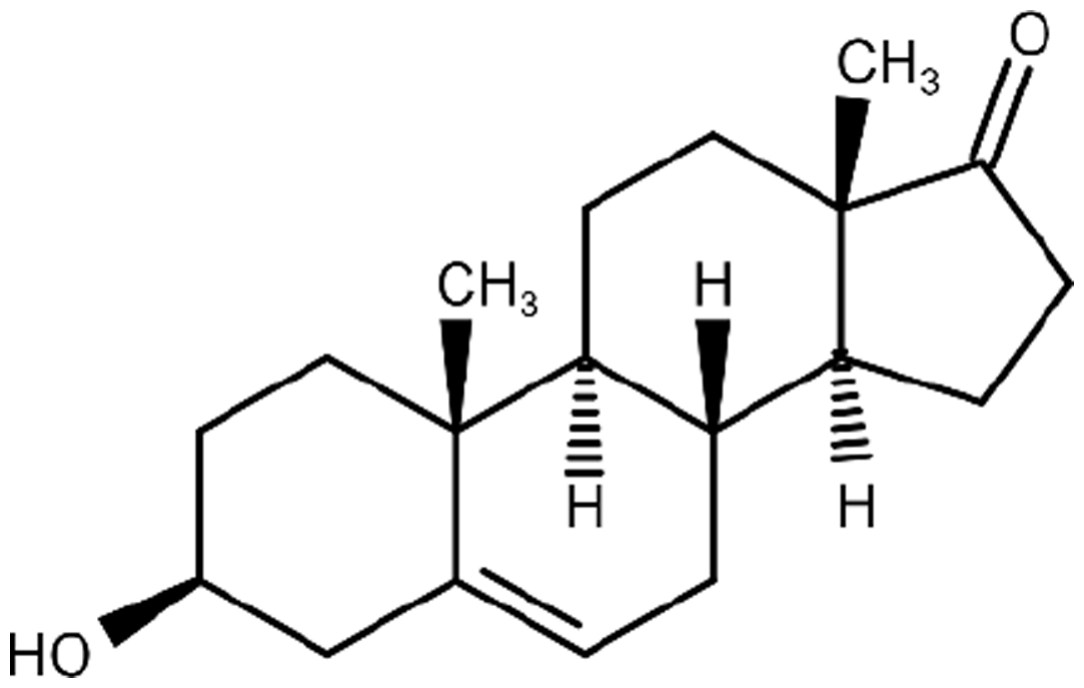
DHEA chemical structure.

**Figure 4:**
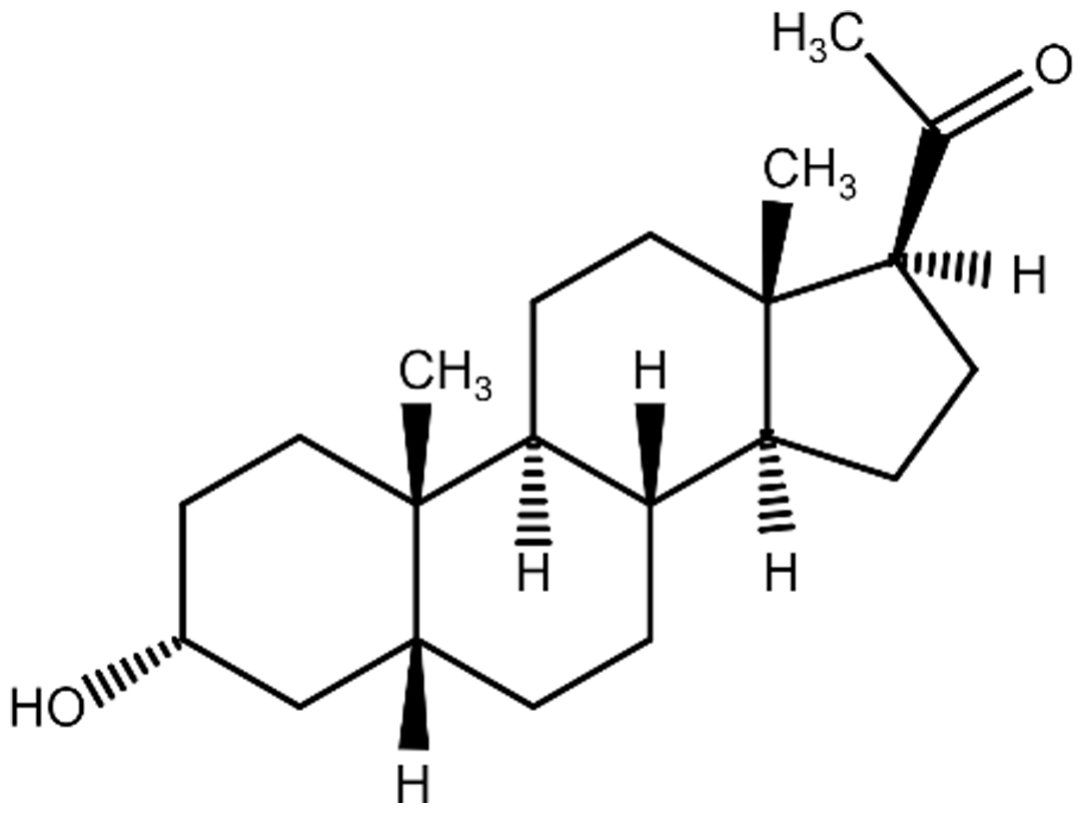
Pregnanolone chemical structure.

To select studies relevant to this review, a literature search was conducted in following databases: the PubMed, Scopus, and Web of Science using the following keywords: ‘zuranolone,’ ‘allopregnanolone,’ ‘brexanolone,’ ‘SAGE-217,’ ‘neurosteroids,’ ‘major depression neurosteroids,’ ‘postpartum depression neurosteroids,’ ‘postnatal psychosis neurosteroids,’ ‘insomnia zuranolone,’ ‘Parkinson’s tremor zuranolone,’ ‘bipolar disorder zuranolone,’ and ‘baby blues.’ Table 4 summarizes screened and included papers (Figures 1–4).

**Table 4:** Summarization of screened and included papers.

**Keyword**	**Screened out studies**	**Included studies**
Zuranolone/SAGE-217	19	6
Brexanolone	54	17
Allopregnanolone	70	20
Neurosteroids	43	12
Major depression neurosteroids	65	5
Schizophrenia neurosteroids	14	2
Postpartum depression neurosteroids	52	21
Postnatal psychosis neurosteroids	4	1
Insomnia zuranolone	2	2
Parkinson’s tremor	1	1
Bipolar disorder zuranolone	1	1
Baby blues	19	0

### 2.1 Inclusion criteria

Studies published since 2017, restricted to adults and related to new neurosteroid treatment agents. Studies explaining the role of certain neurosteroids in the mentioned disorders and were published before and after 2017.

### 2.2 Exclusion criteria

Studies published in a language other than English. Non-peer reviewed literature.

## 3 Review

### 3.1 Zuranolone

SAGE-217 is a synthetic positive allosteric modulator of the GABA_A_ receptor. It is selective for this receptor only (96). Its improved pharmacokinetic properties allow for oral daily dosing. It enhances current in both the γ subunit containing synaptic receptors and the sigma subunit containing extrasynaptic receptors. It affects nine unique human recombinant subtypes in total. It acts synergistically with diazepam in a non-competitive way (96).

To explore the synergy, the method of Tallarida (97) was used to carry out an isobolographic analysis. In simple terms, a specific level of effect (also known as the line of additivity) was selected. The concentrations of diazepam and zuranolone needed to reach this effect when combined were then compared to the concentrations needed for each compound to reach the same effect when used individually (96). According to the findings, a similar therapeutic effect can be achieved with lower dose of diazepam and zuranolone used together than separately (96). This can be beneficial in a strategy of minimalizing adverse effects observed in monotherapy.

In a randomized clinical trial, zuranolone improved HAMD-17 scores in women with PPD and eased symptoms of depression at day 15 when compared to a placebo. Its effects started to appear by day 3 and lasted through all measured time points, including at 45 days. Moreover, patients reported improvements in anxiety and global and maternal functioning (2). In the group of patients with MDD, a 14-day treatment course resulted in a decrease in depressive symptoms scored using the HAM-D score. Comparable results were observed in three phases of trials (4, 98). Table 4 compares Brexanolone and Zuranolone with placebo groups, respectively.

Zuranolone was tested in a randomized, double-blind, placebo-controlled trial involving 543 patients with major depressive disorder. The patients received either zuranolone 50 mg or a placebo once daily for 2 weeks. The trial showed that zuranolone significantly reduced depressive symptoms at day 15, with a fast onset of action by day 33 (10). The drug was generally safe and well tolerated, with no new safety concerns (10). The trial demonstrated the potential of zuranolone as a novel treatment option for adults with major depressive disorder (10).

### 3.2 Zuranolone in comparison with brexanolone

Both zuranolone and brexanolone are neuroactive steroids that selectively modulate the activity of the GAB_A_ receptor. Brexanolone has shown clinical efficacy in patients with postpartum depression and essential tremors. It has also demonstrated anticonvulsant, anxiolytic-like, and antidepressant-like activity in preclinical models. While brexanolone has been more extensively studied and documented, zuranolone has the potential to become a more widely used drug due to its oral bioavailability, while brexanolone requires intravenous administration and constant supervision by healthcare professionals (99). Until recently, it has been challenging to provide the pharmacological benefits of neuroactive steroid GABA modulators to a broad range of patients because of the difficulty of achieving optimal activity at both synaptic and extrasynaptic GABA_A_ receptors while still maintaining a readily available and moderately metabolized profile. This has been especially problematic due to the limited absorption that occurs after oral administration, as well as the quick biotransformation and elimination that follows. As an answer to that, zuranolone was designed through a comprehensive structure–activity-relationship program to optimize the pharmacologic, pharmacokinetic, and pharmacodynamic properties of this class of neuroactive steroid GABA modulators (6, 41, 96, 100–108).

The difference between brexanolone and zuranolone can be verified by the numbers. In a 2020 study, both IP and PO administration of 10 mg/kg zuranolone achieved a maximum plasma concentration (C_max_) at 30 min post-dose. Oral admission resulted in lower plasma C_max_ (1,335 vs. 3,197 ng/mL, respectively) compared to IP administration. The oral bioavailability of zuranolone in mice was moderately high at 62 and 89% after IP admission (96). There is more information about the pharmacokinetics of brexanolone during the standard 60 h infusion. One of the studies has shown that the maximum plasma concentration of brexanolone was achieved at the geometric mean of 47.8 h, and C_max_ was 89.7 ng/mL. Importantly, plasma allopregnanolone concentrations were approaching the LLQQ for all participants by 72 h, which indicates serious problems in the future clinical use of the drug (101, 103, 104, 109–113). Table 5 compares pharmacokinetics of brexanolone and zuranolone and Table 6 compares Brexanolone and Zuranolone with placebo groups, respectively.

**Table 5:** Comparison of pharmacokinetics of brexanolone and zuranolone.

	**Brexanolone IP**	**Zuranolone IP**	**Zuranolone PO**
**Dose**	90 μg/kg/h in 60 h	10 mg/kg	10 mg/kg
**C**_**max**_^**1**^	89.7 ng/mL	3,197 ng/mL	1,335 ng/mL
**t**_**max**_^**2**^	47.8 h	30 min	30 min

BIBLIOGRAPHY \l 1045 ^1^Maximum plasma concentration; ^2^Time to achieve maximum plasma concentration.

**Table 6:** Comparison of brexanolone and zuranolone with placebo groups, respectively.

	**Brexanolone** (104)	**Brexanolone** (104)	**Brexanolone: placebo group** (104)	**Zuranolone** (2)	**Zuranolone: placebo group** (2)
**Mean change from baseline in HAMD-17 score**	–19.5 (SE = 1,2) after 60 h	–17.7 (SE = 1,2) after 60 h	–14.0 (SE = 1,1) after 60 h	–17.8 on day 3	–13.6 on day 3
**Dose and population**	60 μg/kg/h in 60 h IV *n* = 43	90 μg/kg/h in 60 h IV *n* = 41	*n* = 46	30 mg/d IP in 14 days *n* = 77	*n* = 76

### 3.3 Safety

#### 3.3.1 The most frequent adverse effects

According to Deligiannidis et al., Zuranolone was generally well tolerated (2), with most treatment-emergent adverse events being mild or moderate. A total of 275 women were screened and enrolled in the study. Out of these, 153 were selected for randomization, with 76 assigned to the placebo group and 77 to the zuranolone group. However, two patients who were randomized did not receive their doses due to withdrawal (1 from the zuranolone group) and noncompliance (1 from the placebo group). The safety set comprised of 78 patients who were treated with zuranolone and 73 who were treated with placebo. Interestingly, 2 patients who were randomized to receive placebo ended up receiving at least one dose of zuranolone. In the zuranolone group, 76 out of 78 patients (97%) completed the treatment. The reasons for discontinuation of treatment in this group were adverse events (1 patient) and noncompliance (1 patient). The most common adverse effects in the zuranolone group (≥5%) were somnolence, headache, dizziness, upper respiratory tract infection, diarrhea, and sedation (2). The most common adverse effects in the placebo group (≥5%) were headache, somnolence, nausea, dizziness, vomiting, abnormal dreams, and hyperhidrosis (2). No evidence for increased suicidal ideation or suicidal behavior was observed (2).

A study by Bullock et al. from 2020 (*n* = 14) highlights that Zuranolone was generally well tolerated, with no serious adverse events and no adverse events leading to treatment discontinuation reported (9). The most common adverse effects were dizziness, somnolence, and sedation, which occurred in 14.3% of the participants and were mild in severity (9). Two patients reduced their dose of zuranolone due to limb discomfort, salivary hypersecretion, and a confusional state (9).

In another research by Bullock et al. from 2022, Zuranolone was generally well-tolerated, with no serious or severe adverse events, and no discontinuations due to adverse events (5). In this study, which was double-blind and used a three-way crossover design, healthy adults were given either a placebo (41 participants), 30 mg of zuranolone (44 participants), or 45 mg of zuranolone (42 participants) All treatment-emergent adverse events were mild and consistent with the pharmacology of zuranolone and prior studies of zuranolone. The most frequent adverse events (≥2 participants in any period) were headache (placebo, *n* = 2) and fatigue (zuranolone 30 mg, *n* = 2) (5). All other adverse effects were reported by 1 participant each and included dizziness, nausea, somnolence (5). Zuranolone 30 and 45 mg doses did not produce significant next-day effects on sleepiness or psychomotor performance, although more participants from the 45 mg zuranolone treatment group reported signs of sleepiness at the post-polysomnography assessment (5).

According to a recent study by Parikh et al., in the majority of cases patients who were treated with zuranolone + antidepressant therapy (ADT) and experienced treatment-emergent adverse events reported these events as mild or moderate (114). Of the 440 randomized patients, 430 received ≥1 dose of the study drug (zuranolone + ADT, *n* = 212; placebo + ADT, *n* = 218; safety set) during the treatment period; of these, five patients (*n* = 2 and *n* = 3, respectively) prematurely discontinued the study, with no postbaseline efficacy data, leaving a total of 425 patients in the full analysis set (*n* = 210 and *n* = 215, respectively). A total of 186 (87.7%) patients who received zuranolone + ADT and 193 (88.5%) who received placebo + ADT completed treatment; 180 (84.9%) and 177 (81.2%) patients, respectively, completed the study (114). The most frequently observed adverse effects, occurring in at least 10% of patients in either the zuranolone + ADT or placebo + ADT groups, included somnolence, dizziness, headache, and nausea. These findings indicate that zuranolone + ADT led to a quicker alleviation of depressive symptoms compared to placebo + ADT in patients with major depressive disorders. Furthermore, the safety profile of zuranolone + ADT was found to be in line with previous studies (114).

#### 3.3.2 Severe adverse effects

During the aforementioned research by Deligiannidis et al. three patients in each group experienced severe adverse effects, and one patient in each group experienced a serious adverse effect (SAE):a confusional state in the zuranolone group and pancreatitis in the placebo group. One patient in the zuranolone group discontinued because of an adverse effect (intermittent sedation) (2). No notable or clinically significant changes in vital signs, electrocardiograms, or clinical laboratory parameters were reported (2).

The research by. Clayton et al. reported that during the course of treatment, two patients who were administered 30 mg of zuranolone encountered SAEs. In the study, 581 patients were enrolled, with 570 (98.1%) of them receiving at least one dose of the study drug. The drugs administered were zuranolone 20 mg (to 188 patients), zuranolone 30 mg (to 192 patients), and a placebo (to 190 patients). During the 14-day treatment period, less than 10% of the patients stopped treatment, with 4.8% from the zuranolone 20-mg group, 7.8% from the zuranolone 30-mg group, and 7.9% from the placebo group. In total, 157 patients (27.5%) left the study, with the primary reason being withdrawal of consent (15.4% or 88 out of 570), followed by failure to follow-up (6.8%), and adverse events (2.1%). The first patient, who had a long history of major depressive disorder and a previous suicide attempt, attempted suicide again on the fifth day, which might be related to the drug. The second patient, who had a history of bile duct repair, developed a bile duct stone on the second day that required surgical removal. This event was not related to the drug. During the observation period after treatment, SAEs were observed in three patients. The first patient, treated with 30 mg of zuranolone, experienced syncope, and fractures in the ankle, cervical vertebra, and tibia on the 28th day, which were not related to the treatment. The second patient, treated with 20 mg of zuranolone, developed toxic encephalopathy, agitation, delirium, drug abuse, pneumonia, rhabdomyolysis, acute kidney injury, and respiratory failure on the 39th day. These were not related to the treatment and were attributed to cocaine use. The third patient, who received a placebo, had suicidal thoughts on the 22nd day, unrelated to the treatment. Treatment-emergent adverse events that led to discontinuation of treatment were similar across all groups (2.1% in the 30 mg zuranolone group, 1.6% in the 20 mg zuranolone group, and 3.2% in the placebo group). The most common reasons for discontinuation were psychiatric and nervous system disorders. There were no reports of loss of consciousness as a adverse event. There were no significant changes observed in vital signs, clinical laboratory parameters, or electrocardiograms. One patient in the 20 mg zuranolone group passed away during the extended follow-up period of 6 months after discontinuing treatment (on the 142nd day), but this was determined by the investigator to be unrelated to the treatment. The number of patients who reported suicidal thoughts or behaviors, as measured by the Columbia-Suicide Severity Rating Scale (C-SSRS), significantly dropped from the initial assessment across all treatment groups. From the third day of treatment through the final evaluation, less than 13% of patients in any group reported such thoughts or behaviors. On the 182nd day, there was no alteration in the proportion of patients reporting suicidal thoughts or behaviors in the groups treated with 30-mg zuranolone (2.3% [3/131]), 20-mg zuranolone (4.7% [6/127]), or placebo (7.0% [9/129]). The average (SD) change from baseline in Physician Withdrawal Checklist (PWC-20) total scores for the 30-mg zuranolone, 20-mg zuranolone, and placebo groups were –5.7 (7.7), -5.4 (7.5), and –5.4 (7.0), respectively, on day 15, and –6.0 (7.0), –6.1 (7.3), and –5.6 (7.3), respectively, on day 21 (a negative change signifies improvement) (115).

There is limited information available about the safety of zuranolone use during breastfeeding. The concentration of allopregnanolone in breast milk after a 60-h infusion gradually decreased to undetectable levels 3 days after the completion of the infusion. The risk of negative effects on the child is probably low. However, the potential effects of brexanolone and zuranolone on breastfed infants need further investigation (109).

### 3.4 Investigated other usages

#### 3.4.1 Insomnia

It is a common sleep disorder that affects millions of people worldwide. GABA is the primary mediator of inhibitory neurotransmission in the central nervous system and is intimately associated with the regulation of the sleep and wake cycles (116). In a double-blind, three-way crossover study, zuranolone was administrated to patients with insomnia in a single dose of 30 or 45 mg, or a matching placebo. The result showed a significant increase in subjective sleep quality and objective sleep measures. Both zuranolone doses visibly improved the median sleep efficiency, wake after sleep onset, duration of awakenings, and total sleep time, with higher doses having better effects. The study also examined the potential effect of zuranolone on sleep architecture (5). Time spent in the N2 and N3 stages of sleep increased significantly, but there was no significant difference observed in the time spent in the N1 or R stages. Zuranolone was generally well-tolerated, with the most common adverse events being headache and fatigue (5).

#### 3.4.2 Parkinson’s tremor

Parkinson’s disease (PD) is a chronic progressive, neurodegenerative disorder that affects millions of people worldwide. Currently, the primary treatment for the motor symptoms of the illness is dopaminergic therapies like levodopa, carbidopa, MAO-B inhibitors, and COMT inhibitors, which increase dopamine levels to mimic brain dopamine activity (117, 118). However, resting tremor has a variable, unpredictable response to those therapies and is postulated to be mediated by a different pathway than bradykinesia and rigidity. Positive modulation of GABA_A_ receptors is a potential therapeutic target for the treatment of tremors in patients with PD. Zuranolone, with its properties, is being evaluated as an adjunctive treatment for tremor symptoms.

An open-label, exploratory study was conducted, in which 14 patients with PD, who were already receiving stable doses of other medications, received Zuranolone for 7 days and were followed for an additional 7 days after administration of the last dose (9). They were evaluated 12 and 23 h after each dose and 7 days following discontinuation of the drug. The results showed that adjunctive treatment with zuranolone improved tremor symptoms as early as 12 h following the first dose and was sustained throughout the treatment period (9). Similar improvements were noted in PD motor symptoms, evaluated by the MDS-UPDRS Part II total score, as well as in overall experiences of daily living assessed by the MDS-UPDRS Part I (nM-EDL) and Part II (M-EDL) scores. The MDS-UPDRS Part IV subscale measures medication-related motor complications such as dyskinesia, dystonia, and motor fluctuations (ON–OFF) that are associated with long-term dopaminergic treatment (9). The greatest numerical decreases contributing to the change in the Part IV total score were observed for the open-label nature of this study, its short duration, and the limited number of patients, therefore, it is important to carry out more studies in larger populations and for longer durations (9). However, the results of this study are highly promising and give hope for future Zuranolone applications (8, 9, 119–122).

#### 3.4.3 Bipolar disorder

Bipolar disorder is a chronic mental illness characterized by recurrent episodes of mania and depression. Given its chronicity, contribution to disability and morbidity and prevalence of more than 2%, the effective treatment and prevention of the disorder represent an area of significant unmet medical need (123). Zuranolone is also under investigation in patients with bipolar disorder who suffer from depressive symptoms (7, 124, 125).

In an open-label, Phase 2 study, 30 mg of zuranolone was self-administered once daily for 2 weeks, with follow-up through day 42. The primary goal of the study was to examine zuranolone safety and tolerability, but patients’ symptoms were also evaluated using HAMD-17 and MARDS scores. Reductions in both scales were observed from day 3, and the change from baseline at Day 15 was sustained through day 42. Naturally, double-blind, placebo-controlled studies are necessary to evaluate the potential benefit of zuranolone in this condition. However, given the general good tolerability, with no severe or serious Treatment Emergent Adverse Events observed in this study, it seems like zuranolone might be a promising alternative or adjunctive treatment for bipolar disorder patients suffering from depressive symptoms (7, 11, 126, 127).

## 4 Limitations

Included studies did not provide data on whether the patients treated with Zuranolone received psychological support in the form of psychotherapy or psychoeducation.

In the case of neurodegenerative disorders (such as Alzheimer’s disease and HIV-associated dementia), we do not have data about the clinical effects of Zuranolone. As discussed earlier, neurosteroids improve new synaptic connection formation, so Zuranolone could possibly be used in their treatment and/or prevention. This requires further investigation.

Some neoplasms (such as glioblastoma, astrocytoma and oligodendroglioma) are found to be hormone-sensitive for steroids. Research show that this can have both pro-tumorigenic and anti-tumorigenic effect. This creates an opportunity to use drugs like Zuranolone as therapeutic agents, but this needs more data to be collected.

PTSD, schizophrenia and premenstrual dysphoric disorder - mutual feature of this group is decreased concentration of allopregnanolone in CSF. Patients could benefit from administration of Zuranolone, however additional research is obligatory.

Possibility of treatment of postpartum psychosis with progesterone have been already spotted in 1943 in a single case-report (128, 129). It is suspected that a sudden drop in progesterone concentration may cause the onset of PPDs. In this case Zuranolone should find its place in therapy of postnatal psychosis. Further studies would shed more light on this matter.

## 5 Conclusion

Treatment of women during pregnancy and the postpartum period presents multiple challenges. On the one hand, the vast spectrum of biochemical changes in the body unique to pregnancy, and on the other, the limited scope of therapeutics that are safe for the child contributes to these challenges. The rapid development of new methods and substances gives hope for easier and safer therapeutic options in the future.

Thanks to its properties, Zuranolone is promising not only in the treatment of postpartum depressive disorders but also in the treatment of major depression, insomnia, bipolar disorder, and Parkinson’s tremor. It appears that Zuranolone has the potential to be administrated with benzodiazepines like diazepam in order to lower the dose of benzodiazepine and minimize risk of its adverse effects. Zuranolone appears to be a safe substance even during breastfeeding, yet more studies are required before it can be used on a wide scale.

## Author contributions

RM: Writing – original draft, Writing – review & editing, Conceptualization, Investigation. JK: Conceptualization, Investigation, Writing – review & editing. AK: Conceptualization, Investigation, Writing – review & editing. DH: Conceptualization, Investigation, Writing – review & editing. NW: Funding acquisition, Supervision, Writing – review & editing, Investigation.

## Supplementary material

Parts of the figure were drawn by using pictures from Servier Medical Art. Servier Medical Art by Servier is licensed under a Creative Commons Attribution 3.0 Unported License.

## References

[1] Salwan A Maroney M Tremayne L. Patient-reported perceptions of brexanolone in the treatment of postpartum depression: a qualitative analysis. Ment Health Clin. (2023) 12:342–9. doi: 10.9740/MHC.2022.12.342, PMID: 36644587 PMC9819138

[2] Deligiannidis KM Meltzer-Brody S Gunduz-Bruce H Doherty J Jonas J Li S. Effect of Zuranolone vs placebo in postpartum depression: a randomized clinical trial. JAMA Psychiatry. (2021) 78:951–9. doi: 10.1001/jamapsychiatry.2021.1559, PMID: 34190962 PMC8246337

[3] Deligiannidis KM Citrome L Huang MY Acaster S Fridman M Bonthapally V. Effect of Zuranolone on concurrent anxiety and insomnia symptoms in women with postpartum depression. J Clin Psychiatry. (2023) 84. doi: 10.4088/JCP.22m14475, PMID: 36724109

[4] Arnaud A Bonthapally V. Zuranolone provides a rapid response without chronic dosing: response to correspondence by ten Doesschate et al. J Affect Disord. (2022) 301:445–7. doi: 10.1016/j.jad.2022.01.059, PMID: 35032505

[5] Bullock A Gunduz-Bruce H Zammit GK Qin M Li H Sankoh AJ. A phase 1 double-blind, placebo-controlled study of zuranolone (SAGE-217) in a phase advance model of insomnia in healthy adults. Hum Psychopharmacol. (2022) 37. doi: 10.1002/hup.2806, PMID: 34352138 PMC9286466

[6] Epperson CN Rubinow DR Meltzer-Brody S Deligiannidis KM Riesenberg R Krystal AD. Effect of brexanolone on depressive symptoms, anxiety, and insomnia in women with postpartum depression: pooled analyses from 3 double-blind, randomized, placebo-controlled clinical trials in the HUMMINGBIRD clinical program. J Affect Disord. (2023) 320:353–9. doi: 10.1016/j.jad.2022.09.143, PMID: 36191643

[7] Carta MG Bhat KM Preti A. GABAergic neuroactive steroids: a new frontier in bipolar disorders? Behav Brain Funct. (2012) 8:61. doi: 10.1186/1744-9081-8-61, PMID: 23253178 PMC3573983

[8] Di Michele F Luchetti S Bernardi G Romeo E Longone P. Neurosteroid and neurotransmitter alterations in Parkinson’s disease. Front Neuroendocrinol. (2013) 34:132–42. doi: 10.1016/j.yfrne.2013.03.001, PMID: 23563222

[9] Bullock A Kaul I Li S Silber C Doherty J Kanes SJ. Zuranolone as an oral adjunct to treatment of parkinsonian tremor: a phase 2, open-label study. J Neurol Sci. (2021) 421:117277. doi: 10.1016/J.JNS.2020.117277, PMID: 33387701

[10] Clayton AH Lasser R Parikh SV Iosifescu DV Jung JA Kotecha M. Zuranolone for the treatment of adults with major depressive disorder: a randomized, placebo-controlled phase 3 trial. Am J Psychiatry. (2023) 180:676–84. doi: 10.1176/appi.ajp.20220459, PMID: 37132201

[11] Open-label, phase 2 trial of the Oral neuroactive steroid GABAA receptor positive allosteric modulator zuranolone in bipolar disorder I and II. Available at: https://www.hmpgloballearningnetwork.com/site/pcn/posters/open-label-phase-2-trial-oral-neuroactive-steroid-gabaa-receptor-positive-allosteric (Accessed April 15, 2023).

[12] Rai S Pathak A Sharma I. Postpartum psychiatric disorders: early diagnosis and management. Indian J Psychiatry. (2015) 57:S216. doi: 10.4103/0019-5545.161481, PMID: 26330638 PMC4539865

[13] Mughal S Azhar Y Siddiqui W. Postpartum Depression. StatPearls (2022). https://www.ncbi.nlm.nih.gov/books/NBK519070/ (Accessed April 15, 2023).

[14] Luo F Zhu Z Du Y Chen L Cheng Y. Risk factors for postpartum depression based on genetic and epigenetic interactions. Mol Neurobiol. (2023) 60:3979–4003. doi: 10.1007/s12035-023-03313-y, PMID: 37004608

[15] Rudzik AEF Robinson-Smith L Tugwell F Ball HL. Relationships between postpartum depression, sleep, and infant feeding in the early postpartum: an exploratory analysis. Front Psych. (2023) 14:1133386. doi: 10.3389/fpsyt.2023.1133386, PMID: 37032920 PMC10079948

[16] Ding X Liang M Wang H Song Q Guo X Su W. Prenatal stressful life events increase the prevalence of postpartum depression: evidence from prospective cohort studies. J Psychiatr Res. (2023) 160:263–71. doi: 10.1016/j.jpsychires.2023.02.036, PMID: 36889197

[17] Hanach N Radwan H Fakhry R Dennis CL Issa WB MAIE F. Prevalence and risk factors of postpartum depression among women living in the United Arab Emirates. Soc Psychiatry Psychiatr Epidemiol. (2023) 58:395–407. doi: 10.1007/s00127-022-02372-1, PMID: 36239744 PMC9971080

[18] Johansson M Ledung Higgins K Dapi Nzefa L Benderix Y. Postpartum depression and life experiences of mothers with an immigrant background living in the south of Sweden. Int J Qual Stud Health Well-being. (2023) 18:2187333. doi: 10.1080/17482631.2023.2187333 36880807 PMC10013500

[19] Li H Li H Zhong J Wu Q Shen L Tao Z. Association between sleep disorders during pregnancy and risk of postpartum depression: a systematic review and meta-analysis. Arch Womens Ment Health. (2023) 26:259–67. doi: 10.1007/s00737-023-01295-3, PMID: 36738337

[20] Ahmadpour P Faroughi F Mirghafourvand M. The relationship of childbirth experience with postpartum depression and anxiety: a cross-sectional study. BMC Psychol. (2023) 11:58. doi: 10.1186/s40359-023-01105-6, PMID: 36869373 PMC9983514

[21] Yamakawa Y Maruta M Higuchi Y Tokunaga A Iwanaga R Honda S. Factors influencing postpartum depression among Japanese parents: a prospective longitudinal study. Neuropsychopharmacol Rep. (2023) 43:213–21. doi: 10.1002/npr2.12326, PMID: 36915226 PMC10275289

[22] Cheng Z Karra M Guo M Patel V Canning D. Exploring the relationship between Anemia and postpartum depression: evidence from Malawi. Int J Environ Res Public Health. (2023) 20:3178. doi: 10.3390/ijerph20043178, PMID: 36833872 PMC9966145

[23] Oxytocin. Drugs and lactation database (LactMed®) (2023). Available at: https://www.ncbi.nlm.nih.gov/books/NBK501490/ (Accessed April 16, 2023).

[24] Stewart DE Vigod SN. Postpartum depression: pathophysiology, treatment, and emerging therapeutics. Annu Rev Med. (2019) 70:183–96. doi: 10.1146/annurev-med-041217-011106, PMID: 30691372

[25] Payne JL Maguire J. Pathophysiological mechanisms implicated in postpartum depression. Front Neuroendocrinol. (2019) 52:165–80. doi: 10.1016/j.yfrne.2018.12.001, PMID: 30552910 PMC6370514

[26] Sundström-Poromaa I Comasco E Sumner R Luders E. Progesterone - Friend or foe? Front Neuroendocrinol. (2020) 59:100856. doi: 10.1016/j.yfrne.2020.100856, PMID: 32730861

[27] Kang HK John D Bisht B Kaur M Alexis O Worsley A. PROTOCOL: effectiveness of interpersonal psychotherapy in comparison to other psychological and pharmacological interventions for reducing depressive symptoms in women diagnosed with postpartum depression in low and middle-income countries: a systematic review. Campbell Syst Rev. (2020) 16. doi: 10.1002/CL2.1074 PMC8356357 37131982

[28] Rose AL Hopko DR Lejuez CW Magidson JF. Major depressive disorder In: Sturmey P, editor. Functional analysis in clinical treatment. London, United Kingdom: Elsevier Academic Press (2023). 339–73.

[29] Heather R Cecilia C Leyla S Jonaki B Sarra H. Table 9, DSM-IV to DSM-5 major depressive episode/disorder comparison. Substance Abuse and Mental Health Services Administration (US) (2016).

[30] Wesseloo R Kamperman AM Munk-Olsen T Pop VJM Kushner SA Bergink V. Risk of postpartum relapse in bipolar disorder and postpartum psychosis: a systematic review and Meta-analysis. Am J Psychiatry. (2016) 173:117–27. doi: 10.1176/appi.ajp.2015.15010124, PMID: 26514657

[31] Raza SK Raza S. Postpartum Psychosis. StatPearls (2022) Available at: https://www.ncbi.nlm.nih.gov/books/NBK544304/ (Accessed April 16, 2023).

[32] Lewis KJS Di Florio A Forty L Gordon-Smith K Perry A Craddock N. Mania triggered by sleep loss and risk of postpartum psychosis in women with bipolar disorder. J Affect Disord. (2018) 225:624–9. doi: 10.1016/j.jad.2017.08.054, PMID: 28889048

[33] Di Florio A Jones L Forty L Gordon-Smith K Robertson Blackmore E Heron J. Mood disorders and parity - a clue to the aetiology of the postpartum trigger. J Affect Disord. (2014) 152–154:334–9. doi: 10.1016/j.jad.2013.09.034 PMC4025607 24446553

[34] Friedman SH Reed E Ross NE. Postpartum psychosis. Curr Psychiatry Rep. (2023) 25:65–72. doi: 10.1007/s11920-022-01406-4, PMID: 36637712 PMC9838449

[35] Bergink V Rasgon N Wisner KL. Postpartum psychosis: madness, mania, and melancholia in motherhood. Am J Psychiatr. (2016) 173:1179–88. doi: 10.1176/appi.ajp.2016.16040454, PMID: 27609245

[36] Perry A Gordon-Smith K Jones L Jones I. Phenomenology, epidemiology and aetiology of postpartum psychosis: a review. Brain Sci. (2021) 11:1–14. doi: 10.3390/BRAINSCI11010047 PMC7824357 33406713

[37] The global burden of disease 2004. (2023). Available at: https://apps.who.int/iris/handle/10665/43942 (Accessed April 15, 2023).

[38] Weissman MM Klerman GL. Sex differences and the epidemiology of depression. Arch Gen Psychiatry. (1977) 34:98–111. doi: 10.1001/archpsyc.1977.01770130100011, PMID: 319772

[39] Belelli D Lambert JJ Peters JA Gee KW Lan NC. Modulation of human recombinant GABAA receptors by pregnanediols. Neuropharmacology. (1996) 35:1223–31. doi: 10.1016/S0028-3908(96)00066-4, PMID: 9014137

[40] Clark BJ Klinge CM. Structure-function of DHEA binding proteins. Academic Press. (2022). doi: 10.1016/bs.vh.2022.12.002 37717999

[41] Hutcherson TC Cieri-Hutcherson NE Gosciak MF. Brexanolone for postpartum depression. Am J Health-Syst Pharm. (2020) 77:336–45. doi: 10.1093/ajhp/zxz333, PMID: 32073124

[42] Giatti S Garcia-Segura LM Barreto GE Melcangi RC. Neuroactive steroids, neurosteroidogenesis and sex. Prog Neurobiol. (2019) 176:1–17. doi: 10.1016/j.pneurobio.2018.06.007, PMID: 29981391

[43] Hirtz A Rech F Dubois-pot-schneider H Dumond H. Astrocytoma: a hormone-sensitive tumor? Int J Mol Sci. (2020) 21:9114. doi: 10.3390/ijms21239114, PMID: 33266110 PMC7730176

[44] Lin YC Cheung G Porter E Papadopoulos V. The neurosteroid pregnenolone is synthesized by a mitochondrial P450 enzyme other than CYP11A1 in human glial cells. J Biol Chem. (2022) 298:102110. doi: 10.1016/j.jbc.2022.102110, PMID: 35688208 PMC9278081

[45] Guennoun R Labombarda F Gonzalez Deniselle MC Liere P De Nicola AF Schumacher M. Progesterone and allopregnanolone in the central nervous system: response to injury and implication for neuroprotection. J Steroid Biochem Mol Biol. (2015) 146:48–61. doi: 10.1016/j.jsbmb.2014.09.001, PMID: 25196185

[46] Melcangi RC Giatti S Calabrese D Pesaresi M Cermenati G Mitro N. Levels and actions of progesterone and its metabolites in the nervous system during physiological and pathological conditions. Prog Neurobiol. (2014) 113:56–69. doi: 10.1016/j.pneurobio.2013.07.006, PMID: 23958466

[47] Guennoun R. Molecular sciences progesterone in the brain: hormone, neurosteroid and neuroprotectant. Multidisciplinary Digital Publishing Institute. (2020). 10.3390/ijms21155271 PMC7432434 32722286

[48] Gaignard P Savouroux S Liere P Pianos A Thérond P Schumacher M. Effect of sex differences on brain mitochondrial function and its suppression by Ovariectomy and in aged mice. Endocrinology. (2015) 156:2893–904. doi: 10.1210/en.2014-1913, PMID: 26039154

[49] Bixo M Andersson A Winblad B Purdy RH Backstrom T Sweden S. Progesterone, 5a-pregnane-3,20-dione and 3a-hydroxy-5a-pregnane-20-one in specific regions of the human female brain in different endocrine states. Brain Res. (1997) 764:173–8. doi: 10.1016/S0006-8993(97)00455-1, PMID: 9295207

[50] Schüle C Nothdurfter C Rupprecht R. The role of allopregnanolone in depression and anxiety. Prog Neurobiol. (2014) 113:79–87. doi: 10.1016/j.pneurobio.2013.09.003, PMID: 24215796

[51] Mellon SH. Neurosteroid regulation of CNS development. Pharmacol Ther. (2007) 116:107. doi: 10.1016/j.pharmthera.2007.04.011, PMID: 17651807 PMC2386997

[52] Bali A Jaggi AS. Multifunctional aspects of allopregnanolone in stress and related disorders. Prog Neuro-Psychopharmacol Biol Psychiatry. (2014) 48:64–78. doi: 10.1016/J.PNPBP.2013.09.005 24044974

[53] Hollis DM Goetz FW Roberts SB Boyd SK. Acute neurosteroid modulation and subunit isolation of the γ-aminobutyric acidA receptor in the bullfrog, *Rana catesbeiana*. J Mol Endocrinol. (2004) 32:921–34. doi: 10.1677/jme.0.0320921, PMID: 15171722

[54] Verbe J Dubertret C El-Hage W Bonnet-Brilhault F Duriez P. GABAergic approach of postpartum depression: a translational review of literature. Encéphale. (2020) 46:123–34. doi: 10.1016/j.encep.2019.09.007, PMID: 31767256

[55] Alvarez LD Estrin DA. Exploring the molecular basis of neurosteroid binding to the β3 homopentameric GABAA receptor. J Steroid Biochem Mol Biol. (2015) 154:159–67. doi: 10.1016/j.jsbmb.2015.07.012, PMID: 26223009

[56] Schumacher M Mattern C Ghoumari A Oudinet JP Liere P Labombarda F. Revisiting the roles of progesterone and allopregnanolone in the nervous system: resurgence of the progesterone receptors. Prog Neurobiol. (2014) 113:6–39. doi: 10.1016/j.pneurobio.2013.09.004, PMID: 24172649

[57] Hirst JJ Kelleher MA Walker DW Palliser HK. Neuroactive steroids in pregnancy: key regulatory and protective roles in the foetal brain. J Steroid Biochem Mol Biol. (2014) 139:144–53. doi: 10.1016/j.jsbmb.2013.04.002, PMID: 23669456

[58] Melcangi RC Panzica GC. Allopregnanolone: state of the art. Prog Neurobiol. (2014) 113:1–5. doi: 10.1016/j.pneurobio.2013.09.005, PMID: 24121112

[59] Andréen L Nyberg S Turkmen S van Wingen G Fernández G Bäckström T. Sex steroid induced negative mood may be explained by the paradoxical effect mediated by GABAA modulators. Psychoneuroendocrinology. (2009) 34:1121–32. doi: 10.1016/j.psyneuen.2009.02.003, PMID: 19272715

[60] Martinez PE Rubinow DR Nieman LK Koziol DE Morrow AL Schiller CE. 5α-reductase inhibition prevents the luteal phase increase in plasma Allopregnanolone levels and mitigates symptoms in women with premenstrual dysphoric disorder. Neuropsychopharmacology. (2016) 41:1093–102. doi: 10.1038/npp.2015.246, PMID: 26272051 PMC4748434

[61] Almeida FB Pinna G Barros HMT. The role of HPA Axis and Allopregnanolone on the neurobiology of major depressive disorders and PTSD. Int J Mol Sci. (2021) 22:5495. doi: 10.3390/ijms22115495, PMID: 34071053 PMC8197074

[62] Meltzer-Brody S Kanes SJ. Allopregnanolone in postpartum depression: role in pathophysiology and treatment. Neurobiol Stress. (2020) 12:100212. doi: 10.1016/J.YNSTR.2020.100212, PMID: 32435663 PMC7231991

[63] Fruzzetti F Fidecicchi T. Hormonal contraception and depression: updated evidence and implications in clinical practice. Clin Drug Investig. (2020) 40:1097–106. doi: 10.1007/s40261-020-00966-8, PMID: 32980990

[64] Osborne LM Gispen F Sanyal A Yenokyan G Meilman S Payne JL. Lower allopregnanolone during pregnancy predicts postpartum depression: an exploratory study. Psychoneuroendocrinology. (2017) 79:116–21. doi: 10.1016/j.psyneuen.2017.02.012, PMID: 28278440 PMC5420429

[65] Romeo E Ströhle A Spalletta G Di Michele F Hermann B Holsboer F. Effects of antidepressant treatment on neuroactive steroids in major depression. Am J Psychiatry. (1998) 155:910–3. doi: 10.1176/ajp.155.7.910, PMID: 9659856

[66] Standeven LR Osborne LM Betz JF Yenokyan G Voegtline K Hantsoo L. Allopregnanolone and depression and anxiety symptoms across the peripartum: an exploratory study. Arch Womens Ment Health. (2022) 25:521–6. doi: 10.1007/s00737-021-01186-5, PMID: 34714413 PMC9113043

[67] Maguire J. Neuroactive steroids and GABAergic involvement in the neuroendocrine dysfunction associated with major depressive disorder and postpartum depression. Front Cell Neurosci. (2019) 13:83. doi: 10.3389/fncel.2019.00083, PMID: 30906252 PMC6418819

[68] Schüle C Eser D Baghai TC Nothdurfter C Kessler JS Rupprecht R. Neuroactive steroids in affective disorders: target for novel antidepressant or anxiolytic drugs? Neuroscience. (2011) 191:55–77. doi: 10.1016/j.neuroscience.2011.03.025, PMID: 21439354

[69] Rasmusson AM Pinna G Paliwal P Weisman D Gottschalk C Charney D. Decreased cerebrospinal fluid allopregnanolone levels in women with posttraumatic stress disorder. Biol Psychiatry. (2006) 60:704–13. doi: 10.1016/j.biopsych.2006.03.026, PMID: 16934764

[70] Hellgren C Akerud H Skalkidou A Bäckström T Sundström-Poromaa I. Low serum allopregnanolone is associated with symptoms of depression in late pregnancy. Neuropsychobiology. (2014) 69:147–53. doi: 10.1159/000358838, PMID: 24776841

[71] Almeida FB Barros HMT Pinna G. Neurosteroids and neurotrophic factors: what is their promise as biomarkers for major depression and PTSD? Int J Mol Sci. (2021) 22:1–12. doi: 10.3390/IJMS22041758 PMC7916492 33578758

[72] Pineles SL Nillni YI Pinna G Irvine J Webb A Arditte Hall KA. PTSD in women is associated with a block in conversion of progesterone to the GABAergic neurosteroids allopregnanolone and pregnanolone measured in plasma. Psychoneuroendocrinology. (2018) 93:133–41. doi: 10.1016/j.psyneuen.2018.04.024, PMID: 29727810

[73] Rasmusson AM King MW Valovski I Gregor K Scioli-Salter E Pineles SL. Relationships between cerebrospinal fluid GABAergic neurosteroid levels and symptom severity in men with PTSD. Psychoneuroendocrinology. (2019) 102:95–104. doi: 10.1016/j.psyneuen.2018.11.027, PMID: 30529908 PMC6584957

[74] Mellon SH Griffin LD Compagnone NA. Biosynthesis and action of neurosteroids. Brain Res Rev. (2001) 37:3–12. doi: 10.1016/S0165-0173(01)00109-6, PMID: 11744070

[75] Lan NC Gee KW. Neuroactive steroid actions at the GABAA receptor. Horm Behav. (1994) 28:537–44. doi: 10.1006/hbeh.1994.1052, PMID: 7729823

[76] Friedman L Gibbs TT Farb DH. γ-Aminobutyric acid(a) receptor regulation: chronic treatment with pregnanolone uncouples allosteric interactions between steroid and benzodiazepine recognition sites. Mol Pharmacol. (1993) 44:191–7. PMID: 8393520

[77] Deutsch SI Mastropaolo J Hitri A. GABA-active steroids: endogenous modulators of GABA-gated chloride ion conductance. Clin Neuropharmacol. (1992) 15:352–64. doi: 10.1097/00002826-199210000-00002, PMID: 1330306

[78] Eser D Schüle C Baghai TC Romeo E Rupprecht R. Neuroactive steroids in depression and anxiety disorders: clinical studies. Neuroendocrinology. (2007) 84:244–54. doi: 10.1159/000097879 17159334

[79] Ben DR Marx CE Shampine LJ Rubinow DR Schmidt PJ. DHEA metabolism to the neurosteroid androsterone: a possible mechanism of DHEA’s antidepressant action. Psychopharmacology. (2015) 232:3375–83. doi: 10.1007/s00213-015-3991-1, PMID: 26105109 PMC6309885

[80] Tomaselli G Vallée M. Stress and drug abuse-related disorders: the promising therapeutic value of neurosteroids focus on pregnenolone-progesterone-allopregnanolone pathway. Front Neuroendocrinol. (2019) 55:100789. doi: 10.1016/j.yfrne.2019.100789, PMID: 31525393

[81] Rasmusson AM Novikov O Brown KD Pinna G Pineles SL. Pleiotropic endophenotypic and phenotype effects of GABAergic neurosteroid synthesis deficiency in posttraumatic stress disorder. Curr Opin Endocr Metab Res. (2022) 25. doi: 10.1016/j.coemr.2022.100359, PMID: 36909842 PMC10004350

[82] Rasmusson AM Pineles SL Brown KD Pinna G. A role for deficits in GABAergic neurosteroids and their metabolites with NMDA receptor antagonist activity in the pathophysiology of posttraumatic stress disorder. J Neuroendocrinol. (2022) 34:e13062. doi: 10.1111/jne.13062, PMID: 34962690 PMC9233411

[83] Liang JJ Rasmusson AM. Overview of the molecular steps in steroidogenesis of the GABAergic neurosteroids allopregnanolone and pregnanolone. Chronic Stress (Thousand Oaks). (2018) 2:247054701881855. doi: 10.1177/2470547018818555, PMID: 32440589 PMC7219929

[84] Arlt W. Dehydroepiandrosterone and ageing. Best Pract Res Clin Endocrinol Metab. (2004) 18:363–80. doi: 10.1016/j.beem.2004.02.006, PMID: 15261843

[85] Lim JW Lee J Pae AN. Mitochondrial dysfunction and Alzheimer’s disease: prospects for therapeutic intervention. BMB Rep. (2020) 53:47–55. doi: 10.5483/BMBRep.2020.53.1.279, PMID: 31818365 PMC6999825

[86] Porcu P Barron AM Frye CA Walf AA Yang SY He XY. Neurosteroidogenesis today: novel targets for neuroactive steroid synthesis and action and their relevance for translational research. J Neuroendocrinol. (2016) 28:1–19. doi: 10.1111/JNE.12351 PMC4769676 26681259

[87] Parks EE Logan S Yeganeh A Farley JA Owen DB Sonntag WE. Interleukin 6 reduces allopregnanolone synthesis in the brain and contributes to age-related cognitive decline in mice. J Lipid Res. (2020) 61:1308–19. doi: 10.1194/jlr.RA119000479, PMID: 32669383 PMC7529050

[88] Brinton RD. Neurosteroids as regenerative agents in the brain: therapeutic implications. Nat Rev Endocrinol. (2013) 9:241–50. doi: 10.1038/nrendo.2013.31, PMID: 23438839 PMC9936614

[89] Février M Dorgham K Rebollo A. CD4 + T cell depletion in human immunodeficiency virus (HIV) infection: role of apoptosis. Viruses. (2011) 3:586–612. doi: 10.3390/v3050586, PMID: 21994747 PMC3185763

[90] Saylor D Dickens AM Sacktor N Haughey N Slusher B Pletnikov M. HIV-associated neurocognitive disorder-pathogenesis and prospects for treatment HHS public access. Nat Rev Neurol. (2016) 12:234–48. doi: 10.1038/nrneurol.2016.27, PMID: 26965674 PMC4937456

[91] Baulieu EE. NEUROSTEROIDS: a novel function of the BRAIN. Psychoneuroendocrinology. (1998) 23:963–87. doi: 10.1016/S0306-4530(98)00071-7, PMID: 9924747

[92] Gursoy E Cardounel A Kalimi M. Pregnenolone protects mouse hippocampal (HT-22) cells against glutamate and amyloid beta protein toxicity. Neurochem Res. (2001) 26:15–21. doi: 10.1023/A:1007668213330, PMID: 11358277

[93] Bhagavathi Perumal M Dhanasekaran S. HIV associated dementia: role for neurosteroids. Med Hypotheses. (2012) 78:672–4. doi: 10.1016/j.mehy.2012.02.008, PMID: 22386322

[94] Pinacho-Garcia LM Valdez RA Navarrete A Cabeza M Segovia J Romano MC. The effect of finasteride and dutasteride on the synthesis of neurosteroids by glioblastoma cells. Steroids. (2020) 155:108556. doi: 10.1016/j.steroids.2019.108556, PMID: 31866547

[95] Seol HJ Kim JE Wang KC Kim SK Seo JS Park SH. The pattern of gene expression and possible relation of steroidogenic genes in oligodendroglial tumors. Int J Oncol. (2009) 34:181–90. doi: 10.3892/ijo_00000140 PMID: 19082489

[96] Althaus AL Ackley MA Belfort GM Gee SM Dai J Nguyen DP. Preclinical characterization of zuranolone (SAGE-217), a selective neuroactive steroid GABAA receptor positive allosteric modulator. Neuropharmacology. (2020) 181. doi: 10.1016/j.neuropharm.2020.108333, PMID: 32976892 PMC8265595

[97] Tallarida RJ. Quantitative methods for assessing drug synergism. Genes Cancer. (2011) 2:1003–8. doi: 10.1177/1947601912440575 22737266 PMC3379564

[98] Gunduz-Bruce H Silber C Kaul I Rothschild AJ Riesenberg R Sankoh AJ. Trial of SAGE-217 in patients with major depressive disorder. N Engl J Med. (2019) 381:903–11. doi: 10.1056/NEJMoa1815981, PMID: 31483961

[99] What is ZULRESSO®? | ZULRESSO® (brexanolone) CIV. (2023). Available at: https://www.zulresso.com/about-zulresso (Accessed April 15, 2023).

[100] Azhar Y Din AU. Brexanolone. Prescribers Guide. (2022):99–102. doi: 10.1017/9781108921275.017

[101] Powell JG Garland S Preston K Piszczatoski C. Brexanolone (Zulresso): finally, an FDA-approved treatment for postpartum depression. Ann Pharmacother. (2020) 54:157–63. doi: 10.1177/1060028019873320, PMID: 31476884

[102] Phillips K Susser LC. Clinical implications of the neurosteroid allopregnanolone in reproductive depression. Harv Rev Psychiatry. (2023) 31:37–45. doi: 10.1097/HRP.0000000000000354 36608082

[103] Edinoff AN Odisho AS Lewis K Kaskas A Hunt G Cornett EM. Brexanolone, a GABAA modulator, in the treatment of postpartum depression in adults: a comprehensive review. Front Psych. (2021) 12:699740. doi: 10.3389/fpsyt.2021.699740, PMID: 34594247 PMC8477036

[104] Meltzer-Brody S Colquhoun H Riesenberg R Epperson CN Deligiannidis KM Rubinow DR. Brexanolone injection in post-partum depression: two multicentre, double-blind, randomised, placebo-controlled, phase 3 trials. Lancet. (2018) 392:1058–70. doi: 10.1016/S0140-6736(18)31551-4, PMID: 30177236

[105] Ali M Aamir A Diwan MN Awan HA Ullah I Irfan M. Treating postpartum depression: what do we know about Brexanolone? Diseases. (2021) 9:52. doi: 10.3390/diseases9030052, PMID: 34287271 PMC8293057

[106] Cooper MC Kilvert HS Hodgkins P Roskell NS Eldar-Lissai A. Using matching-adjusted indirect comparisons and network Meta-analyses to compare efficacy of Brexanolone injection with selective serotonin reuptake inhibitors for treating postpartum depression. CNS Drugs. (2019) 33:1039–52. doi: 10.1007/s40263-019-00672-w, PMID: 31642037 PMC6825025

[107] Leader LD O’Connell M VandenBerg A. Brexanolone for postpartum depression: clinical evidence and practical considerations. Pharmacotherapy. (2019) 39:1105–12. doi: 10.1002/phar.2331, PMID: 31514247

[108] Gunduz-Bruce H Takahashi K Huang MY. Development of neuroactive steroids for the treatment of postpartum depression. J Neuroendocrinol. (2022) 34:e13019. doi: 10.1111/jne.13019, PMID: 34462985 PMC9285576

[109] Wald J Henningsson A Hanze E Hoffmann E Li H Colquhoun H. Allopregnanolone concentrations in breast Milk and plasma from healthy volunteers receiving Brexanolone injection, with population pharmacokinetic modeling of potential relative infant dose. Clin Pharmacokinet. (2022) 61:1307–19. doi: 10.1007/s40262-022-01155-w, PMID: 35869362 PMC9439988

[110] Kanes S Colquhoun H Gunduz-Bruce H Raines S Arnold R Schacterle A. Brexanolone (SAGE-547 injection) in post-partum depression: a randomised controlled trial. Lancet. (2017) 390:480–9. doi: 10.1016/S0140-6736(17)31264-3, PMID: 28619476

[111] Walkery A Leader LD Cooke E Vandenberg A. Review of Allopregnanolone agonist therapy for the treatment of depressive disorders. Drug Des Devel Ther. (2021) 15:3017–26. doi: 10.2147/DDDT.S240856 PMC8276990 34267503

[112] Zheng W Bin CD Sim K Ungvari GS Peng XJ Ning YP. Brexanolone for postpartum depression: a meta-analysis of randomized controlled studies. Psychiatry Res. (2019) 279:83–9. doi: 10.1016/j.psychres.2019.07.006, PMID: 31323375

[113] Faden J Citrome L. Intravenous brexanolone for postpartum depression: what it is, how well does it work, and will it be used? Ther Adv Psychopharmacol. (2020) 10:204512532096865. doi: 10.1177/2045125320968658, PMID: 33224470 PMC7656877

[114] Parikh SV Aaronson ST Mathew SJ Alva G DeBattista C Kanes S. Efficacy and safety of zuranolone co-initiated with an antidepressant in adults with major depressive disorder: results from the phase 3 CORAL study. Neuropsychopharmacology. (2023) 2023:1–9. doi: 10.1038/s41386-023-01751-9 PMC10724299 37875578

[115] Clayton AH Lasser R Nandy I Sankoh AJ Jonas J Kanes SJ. Zuranolone in major depressive disorder: results from MOUNTAIN—A phase 3, multicenter, double-blind, randomized, placebo-controlled trial. J Clin Psychiatry. (2023) 84:45750. doi: 10.4088/JCP.22M14445 36811520

[116] Wisden W Yu X Franks NP. GABA receptors and the pharmacology of sleep. Handb Exp Pharmacol. (2019) 253:279–304. doi: 10.1007/164_2017_56 28993837

[117] Fox SH Katzenschlager R Lim SY Barton B de Bie RMA Seppi K. International Parkinson and movement disorder society evidence-based medicine review: update on treatments for the motor symptoms of Parkinson’s disease. Mov Disord. (2018) 33:1248–66. doi: 10.1002/mds.27372, PMID: 29570866

[118] Reich SG Savitt JM. Parkinson’s disease. Med Clin North Am. (2019) 103:337–50. doi: 10.1016/j.mcna.2018.10.014, PMID: 30704685

[119] Yilmaz C Karali K Fodelianaki G Gravanis A Chavakis T Charalampopoulos I. Neurosteroids as regulators of neuroinflammation. Front Neuroendocrinol. (2019) 55. doi: 10.1016/j.yfrne.2019.100788, PMID: 31513776

[120] Lloyd-Evans E Waller-Evans H. Biosynthesis and signalling functions of central and peripheral nervous system neurosteroids in health and disease. Essays Biochem. (2020) 64:591–606. doi: 10.1042/EBC20200043, PMID: 32756865 PMC7517341

[121] Luchetti S Huitinga I Swaab DF. Neurosteroid and GABA-A receptor alterations in Alzheimer’s disease, Parkinson’s disease and multiple sclerosis. Neuroscience. (2011) 191:6–21. doi: 10.1016/j.neuroscience.2011.04.010, PMID: 21514366

[122] Nezhadi A Sheibani V Esmaeilpour K Shabani M Esmaeili-Mahani S. Neurosteroid allopregnanolone attenuates cognitive dysfunctions in 6-OHDA-induced rat model of Parkinson’s disease. Behav Brain Res. (2016) 305:258–64. doi: 10.1016/j.bbr.2016.03.019, PMID: 26970579

[123] Haggarty SJ Karmacharya R Perlis RH. Advances toward precision medicine for bipolar disorder: mechanisms & molecules. Mol Psychiatry. (2021) 26:168–85. doi: 10.1038/s41380-020-0831-4, PMID: 32636474 PMC10290523

[124] Brown ES Park J Marx CE Hynan LS Gardner C Davila D. A randomized, double-blind, placebo-controlled trial of pregnenolone for bipolar depression. Neuropsychopharmacology. (2014) 39:2867–73. doi: 10.1038/npp.2014.138, PMID: 24917198 PMC4200497

[125] Mason BL Van Enkevort E Filbey F Marx CE Park J Nakamura A. Neurosteroid levels in patients with bipolar disorder and a history of Cannabis use disorders. J Clin Psychopharmacol. (2017) 37:684–8. doi: 10.1097/JCP.0000000000000793, PMID: 29045302

[126] Youssef NA Bradford DW Kilts JD Szabo ST Naylor JC Allen TB. Exploratory investigation of biomarker candidates for suicide in schizophrenia and bipolar disorder. Crisis. (2015) 36:46–54. doi: 10.1027/0227-5910/a000280, PMID: 25410258

[127] Marx CE Stevens RD Shampine LJ Uzunova V Trost WT Butterfield MI. Neuroactive steroids are altered in schizophrenia and bipolar disorder: relevance to pathophysiology and therapeutics. Neuropsychopharmacology. (2006) 31:1249–63. doi: 10.1038/sj.npp.1300952, PMID: 16319920

[128] Schmidt HJ. The use of progesterone in the treatment of postpartum psychosis. J Am Med Assoc. (1943) 121:190–2. doi: 10.1001/jama.1943.02840030028007, PMID: 34594247

[129] Rupprecht R Koch M Montkowski A Lancel M Faulhaber J Harting J. Assessment of neuroleptic-like properties of progesterone. Psychopharmacology. (1999) 143:29–38. doi: 10.1007/s002130050916, PMID: 10227077

