# Supplementary figures and images for: Zuranolone – synthetic neurosteroid in treatment of mental disorders: narrative review
Source: Front Psychiatry. 2023 Dec 5;14:1298359. doi: 10.3389/fpsyt.2023.1298359 (PMC10729607; doi:10.3389/fpsyt.2023.1298359)

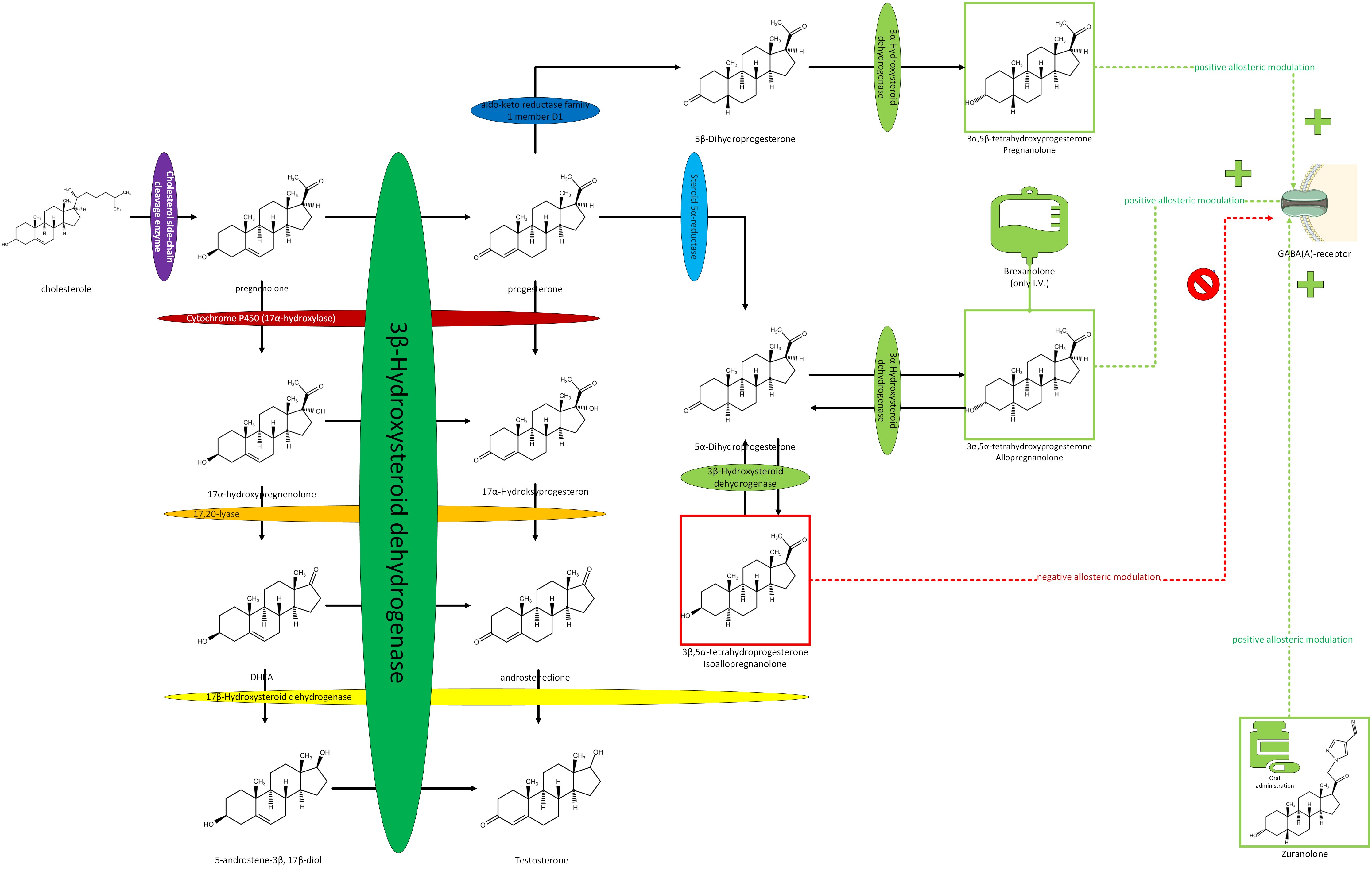

Supplement: Supplementary file 1 [file Image_1.JPEG]
